# Optimizing a qPCR Gene Expression Quantification Assay for *S. epidermidis* Biofilms: A Comparison between Commercial Kits and a Customized Protocol

**DOI:** 10.1371/journal.pone.0037480

**Published:** 2012-05-21

**Authors:** Angela França, Ana I. Freitas, Ana F. Henriques, Nuno Cerca

**Affiliations:** CEB-IBB, Centro de Engenharia Biológica - Instituto de Biotecnologia e Bioengenharia, Campus de Gualtar, Universidade do Minho, Braga, Portugal; University of Padova, Italy

## Abstract

*Staphylococcus epidermidis* biofilm-related infections are a current concern within the medical community due to their high incidence and prevalence, particularly in patients with indwelling medical devices. Biofilm gene expression analysis by quantitative real-time PCR (qPCR) has been increasingly used to understand the role of biofilm formation in the pathogenesis of *S. epidermidis* infections. However, depending on the RNA extraction procedure, and cDNA synthesis and qPCR master mixes used, gene expression quantification can be suboptimal. We recently showed that some RNA extraction kits are not suitable for *S. epidermidis* biofilms, due to sample composition, in particular the presence of the extracellular matrix. In this work, we describe a custom RNA extraction assay followed by the evaluation of gene expression using different commercial reverse transcriptase kits and qPCR master mixes. Our custom RNA extraction assay was able to produce good quality RNA with reproducible gene expression quantification, reducing the time and the costs associated. We also tested the effect of reducing cDNA and qPCR reaction volumes and, in most of the cases tested, no significant differences were found. Finally, we titered the SYBR Green I concentrations in standard PCR master mixes and compared the normalized expression of the genes *icaA*, *bhp*, *aap*, *psmβ1* and *agrB* using 4 distinct biofilm forming *S. epidermidis* strains to the results obtained with commercially available kits. The overall results demonstrated that despite some statistically, but not biologically significant differences observed, the customized qPCR protocol resulted in the same gene expression trend presented by the commercially available kits used.

## Introduction

*Staphylococcus epidermidis*, a commensal inhabitant of human skin and mucosa is a leading cause of nosocomial infections, particularly, in patients with indwelling medical devices [1]. The emergence and pathogenesis of S. *epidermidis* infections are related with its ability to adhere and form biofilms on the surface of those medical devices [2]. Biofilms, 3-dimensional and structured communities of bacteria enclosed in a matrix of extra-polymeric substances, that include polysaccharides, proteins, lipids and extracellular DNA, have been shown to protect bacteria against antibiotics therapy and host immune system attack [3], [4]. This frequently results in the development of recalcitrant chronic infections [5]. Therefore, *S. epidermidis* biofilm-related infections are associated with significant morbidity and occasionally, mortality. These infections also present a significant financial burden and it was estimated that annually, in the United States alone, these infections increase health costs by $2 billion U.S. dollars [2].

In the last few years, several research groups have been studying the molecular mechanisms behind *S. epidermidis* biofilm formation and resistance [6]–[10]. The quantification of specific messenger RNA (mRNA) has proved to be a useful tool to validate the transcriptional measurements associated with switching to the pathogenic mode of infection [11]–[14]. Nonetheless, the success of any RNA-based analysis relies on the quality of the mRNA, since the purity and integrity of this molecule can impact the accuracy of processing or analytic techniques [15], [16] such as complementary DNA (cDNA) synthesis and quantitative real-time PCR (qPCR) or genome-wide analysis such as DNA microarrays or RNA sequencing. Currently, genome-wide analyses have been increasingly used for gene expression profiling analysis while qPCR is regularly used to study the expression of specific set of genes. Additionally, qPCR is considered the gold standard technique to validate genome-wide analysis results [17], [18] and therefore, despite the limitation regarding the number of genes that can be analyzed at each time, qPCR is still a widely used technique.

Currently, there are several commercially available kits for RNA extraction, cDNA synthesis and gene expression quantification by qPCR. However, most of these kits were not tested in biofilm cultures, and depending on the principle and properties of each kit, the accuracy of the mRNA transcripts quantification can be impaired [19], [20]. Therefore, in this work we aimed to compare different commercially available kits and, simultaneously, optimize a customized protocol for gene expression quantification by qPCR using *S. epidermidis* biofilm as samples. The custom protocol was optimized to maximize reliability of results, reduce time, and minimize costs involved.

## Materials and Methods

### Bacterial strains and growth conditions

The *S. epidermidis* strains used in this work were previously characterized regarding biofilm formation capacity: 9142, LE7, IE186 and M129 [21]. Biofilms were formed as described [4]. Briefly, a single colony of each *S. epidermidis* strains used was inoculated in Tryptic Soy Broth (TSB) (Oxoid, Cambridge, UK) and incubated at 37°C in a shaker at 120 rpm overnight. Afterwards, 1 100 dilution was performed in fresh TSB supplemented with 1% (w/v) of glucose (Fisher Scientific, Waltham, MA, US) to induce biofilm formation in a 24-well plate (Orange Scientific, Braine-l'Alleud, Belgium) and incubated in the same conditions for 24±2 hours. Biofilms were then washed and resuspended in 1 mL of 0.9% NaCl. Planktonic bacteria were grown in 2 mL TSB in 15 mL falcon tubes at 37°C in a shaker at 120 rpm for 18±1 hours.

### RNA extraction with commercially available kits

Based on our previous findings [19] we selected two commercially available kits with distinct principles: FastRNA® Pro Blue (MPBiomedicals, Irvine, CA, US) that uses mechanical and chemical lysis together with organic extraction and PureLink™ RNA Mini Kit (Invitrogen, San Diego, CA, US) that uses enzymatic lysis and silica membrane extraction. Total RNA was isolated according to the manufacturers' instructions, with the following optimization: when appropriate cell lysis was performed using 15 mg/mL of lysozyme (Sigma, St Louis, MO, US) for 60 min at 37°C with. This optimization increased the yield of total RNA 2 to 4-fold (data not shown).

### Customized RNA extraction protocol

The following protocol was optimized based on the mechanical and chemical lysis required to effectively extract RNA from *S. epidermidis* biofilms [19] and on subsequent silica membrane isolation, in order to reduce the time needed for RNA extraction. The protocol described here used the ISOLATE RNA Mini kit columns systems (Bioline, London, UK). However, we tested the same approach with other column systems such as PureLink™ RNA Mini Kit (Invitrogen), Direct-zol™ RNA MiniPrep (Zymo Research, Irvine, CA, USA) and FavorPrep™ Blood/Cultured Cell Total RNA (Favorgen, Ping-Tung, Taiwan) obtaining similar results.

Briefly, bacteria were resuspended in 100 µL RNase free water and transferred to a 2 mL safe lock tube containing 0.4 g of acid-washed 150–212 mm silica beads (Sigma), 400 µL Lysis buffer R (provided by the kit) and 400 µL 90% phenol solution (AppliChem, Darmstadt, Germany). This mixture was vortexed for 20 seconds before using the FastPrep® cell disruptor (BIO 101, ThermoElectron Corporation, Thermo Scientific) with setting 6.5 for 35 seconds. The samples were then cooled on ice and the beat-beading step repeated twice. Afterwards, samples were centrifuged at 16,000 *g* for 5 minutes and supernatants transferred to a new tube and mixed with equal volume of 100% ethanol (Fisher Scientific). The samples (including any remaining precipitate) were transferred to the silica columns and centrifuged at 12,000 *g* for 15 seconds at room temperature (RT). The flow-through was discarded and each column was reinserted into a new collection tube. To wash the columns, 700 µL of Wash buffer I was added to each column and centrifuged at 12,000 *g* for 15 seconds at RT. The flow-through was discarded and inserted into the same collection tube. After that, 500 µL of Wash buffer II was added to each column and centrifuged at 12,000 *g* for 15 seconds at RT. The flow-through was discarded and the columns reinsert into a new collection tube for a new centrifugation at 12,000 *g* for 2 minutes. The collection tube was discarded and each column was inserted into a recovery tube. Finally, RNA elution was achieved by adding 45 µL of RNase-free water to the center of the membrane, incubated for 1 minute and centrifuged for 1 minute at 12,000 *g*. All steps were done at room temperature, except where otherwise noticed.

### DNase treatment

Genomic DNA was digested with DNase I (Fermentas, Ontario, Canada). Briefly, 2 µL of DNase I and 5 µL of reaction buffer were added to the RNA sample and incubated at 37°C for 30 minutes. Then, to inactivate the DNase I enzyme, 5 µL of 25 mM EDTA was added to the mixture and incubated at 65°C for 10 minutes.

### RNA quality determination

The concentration and purity of the total RNA was spectrometrically determined using a NanoDrop 1000™ (Thermo Scientific). Before measuring the RNA, the nanodrop was activated and the light source was allowed to warm up and stabilize. Three independent measurements of the same sample were performed. The absorbance ratio A_260_/_A280_ was used as an indicator of protein contamination and A_260_/A_230_ as an indicator of polysaccharide, phenol, and/or chaotropic salt contamination [22]. The integrity of the total RNA was assessed by visualization of the 23S/16S banding pattern. Electrophoresis was carried out at 80 V for 60 minutes using a 1.5% agarose gel. The gel was stained with ethidium bromide and visualized using a GelDoc2000 (Bio-Rad, Hercules, CA, US). RNA was stored at −80°C for further use.

### cDNA synthesis

cDNA synthesis was performed using 4 different commercial kits: Super Script® VILO™ (Invitrogen), RevertAaid™ First Strand cDNA Synthesis kit (Fermentas), iScript™ cDNA synthesis (Bio-Rad) and qScript™ cDNA Synthesis (Quanta BioSciences, Gaithersburg, MD, US) following the manufacturer's instructions. The same amount of total RNA (500 ng/20 µL) was reverse transcribed in two reaction volumes: 20 µL, as described by the manufacturer, and 10 µL. To determine the possibility of genomic DNA carry-over, control reactions were performed under the same conditions but lacking the reverse transcriptase enzyme (no-RT control). All RNA extracted was absent of significant genomic DNA, as determined by an average cycle threshold difference of 18.5±3.5 , equivalent to a maximum quantification error of 0.0003%.

### Gene expression quantification

Biofilm gene expression was determined by qPCR. Oligonucleotide primers for the detection of 16S rRNA, *icaA*, *aap, bh* and *psmβ1 and agrB* were designed using the Primer3 software [23] having either *S. epidermidis* RP62A (PubMed accession number NC_002976.3) or ATCC12228 (PubMed accession number NC_004461.1) genome, respectively, as template (Table S1). qPCR analysis was performed using 4 different commercial qPCR master mixes (mi-real-time EvaGreen® Master (Metabion, Martinsried, Germany), Maxima® SYBR Green Master Mix (Fermentas), iQ™ SYBR® Green Supermix (Bio-Rad) and PerfeCTa® SYBR® Green SuperMix (Quanta BioSciences)) and also by using 3 standard PCR kits based on Taq polymerase (DyNAzyme™ II PCR Master Mix (Finzymes, Vantaa, Finland), MyTaq PCR mix (Bioline, London, UK) or EzWay Direct Taq PCR MasterMix (Koma Biotech, Seoul, South Korea)). For transcripts detection, SYBR Green I (Invitrogen) was added to a standard PCR mix, at different concentrations, ranging from 3.2× to 0.1×. Two reaction volumes were tested: 20 and 10 µL. The 20 µL reactions contained 2 µL diluted cDNA or no-RT control, 10 pmol of each primer, 6 µL nuclease free H_2_O, and 10 µL of the respective 2× master mix. The 10 µL reactions contained half the respective volumes. Primer efficiencies were determined by the dilution method as well as performing a temperature gradient reaction from 50 to 65°C [19]. At 60°C, both set of primers had the best and more similar efficiencies values. qPCR run was performed on a CFX 96 (Bio-Rad) with the following cycle parameter: 95°C for 30 s, 39 cycles of 95°C for 5 s, 60°C for 15 s and 68°C for 15 s. qPCR products were analyzed by melting curves for unspecific products or primer dimer formation. Relative fold increase of specific mRNA transcripts in biofilms comparing with planktonic cultures, was calculated using 2^ΔCt^ method, a variation of the Livak method, where 2 stands for the 100% reaction efficiency (the reaction efficiency was determined experimentally and thus 100% efficiency was replace by the real efficiency) and ΔCt = Ct (housekeeping gene)-Ct (target gene). The data analysis was based on at least 3 independent experiments.

### Statistical analysis

All the assays were compared using one-way analysis of variance (ANOVA) by applying Levene's test of homogeneity of variances and Tukey's multiple comparisons test, and also the paired sample t-test, using SPSS. Student's t-test was applied to all experimental data for rejection of some experimental values. All tests were performed with a confidence level of 95%.

## Results

### RNA extraction

Based on our previous findings [19] we selected two commercially available RNA extraction kits with distinct principles: FastRNA® Pro Blue, which uses mechanical and chemical lysis together with organic extraction, and PureLink™ RNA Mini Kit, which uses enzymatic lysis and silica-based membrane extraction. We then combined the best features of both kits, namely the high yield resulting from the glass beads- and phenol-based lysis and the fast isolation protocol provided by the columns system, as described in the material and methods section. For the custom extraction, we tested 4 different column-based isolation kits. As illustrated by the results in Table 1, the PureLink™ kit yielded very low concentration of RNA. However, when PureLink™ column system was combined with the custom lysis, we were able to recover 26-fold more total RNA, obtaining very similar values as that obtained when using the Fast RNA® Pro Blue kit. All the other columns system tested resulted in high RNA yield. Besides yield, other common extraction performance indicators are RNA purity and integrity [15]. The absorbance ratios A_260_/A_280_ were used as indicators of protein contamination and A_260_/A_230_ as indicators of polysaccharide, phenol, and/or chaotropic salt contamination [22]. The referred absorbance ratios should be above 1.8, in order to have pure RNA [24]. While all RNA extraction procedures resulted in acceptably low levels of protein contamination (A_260_/A_280_>1.8), some of the kits presented an A_260_/A_230_ below 1.8. The integrity of the total RNA was assessed by visualization of the 23S/16S banding pattern. RNA from all extraction procedures was intact (Figure S1). No integrity information was determined for the RNA extracted with PureLink™ RNA Mini Kit, as the low yield was below the limit of detection of our image system, as described elsewhere [19], [20].

**Table 1:** Comparison of RNA yield and purity obtained by the different RNA extraction procedures used.

RNA extraction kit	RNA yield (ng/µL)	A_260_/A_280_ ratio	A_260_/A_230_ ratio
FastRNA®	499±74	2,2±0,0	2,1±0,1*
PureLink™	17±3*	2,0±0,1	1,4±0,2*
Custom RNA w/PureLink™	453±49	2,0±0,1	1,4±0,6*
Custom RNA w/FavorPrep™	226±31*	1,8±0,1*	1,8±0,2*
Custom RNA w/Direct-zol™	182±5*	2,1±0,1	2,2±0,2*
Custom RNA w/RNA Mini spin	422±84	1,9±0,1	1,6±0,1*

24 H biofilms of *S. epidermidis* were disrupted and RNA extraction performed using commercially available kits or an optimized custom procedure. The values represent the mean plus or minus the standard deviation of 3 independent experiments. Statistical differences between custom and commercial kits (*p<0.01) were analyzed with paired t-test.

### cDNA and qPCR reaction optimization

**Figure 1:**
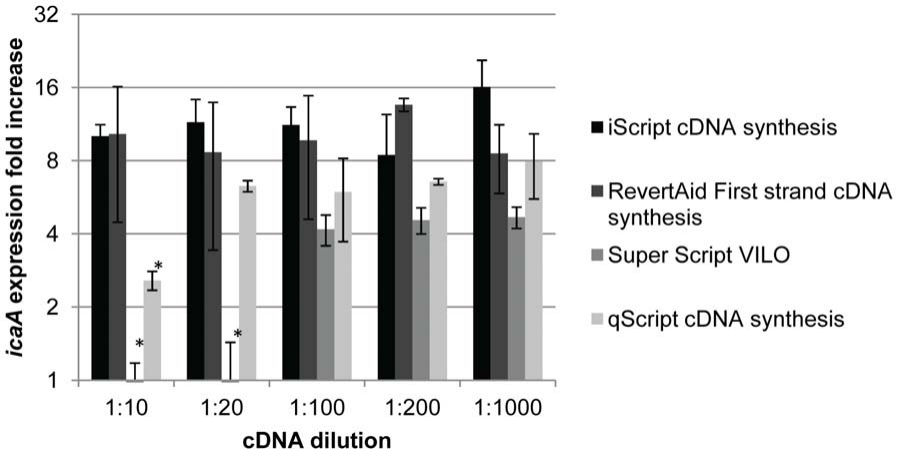
Effect of cDNA dilution in *icaA* gene expression quantification. cDNA from biofilms or planktonic cultures was diluted from 1 10 to 1 1000 fold and *icaA* transcripts quantified by qPCR. cDNA replicates were synthesized using the same RNA sample but in independent cDNA synthesis reactions. The values represent the mean plus or minus standard deviation of 3 independent experiments. Statistical differences (*p<0.05) were analyzed with ANOVA Tukey's test.

In qPCR a common and important optimization step is the determination of the optimal complementary DNA (cDNA) dilution that should be used in order to obtain reproducible and meaningful results. Undiluted cDNA can interfere with the PCR reaction and, thus, several cDNA dilutions were tested by determining the *icaA* gene fold increase in biofilms samples (Figure 1). The lowest dilution common to all the 4 tested kits that shown reliable results, as assessed by no significant variation between the tested cDNA concentrations, was the 1 100 dilution. Therefore, for all the further studies, cDNA was diluted 100 fold.

**Figure 2:**
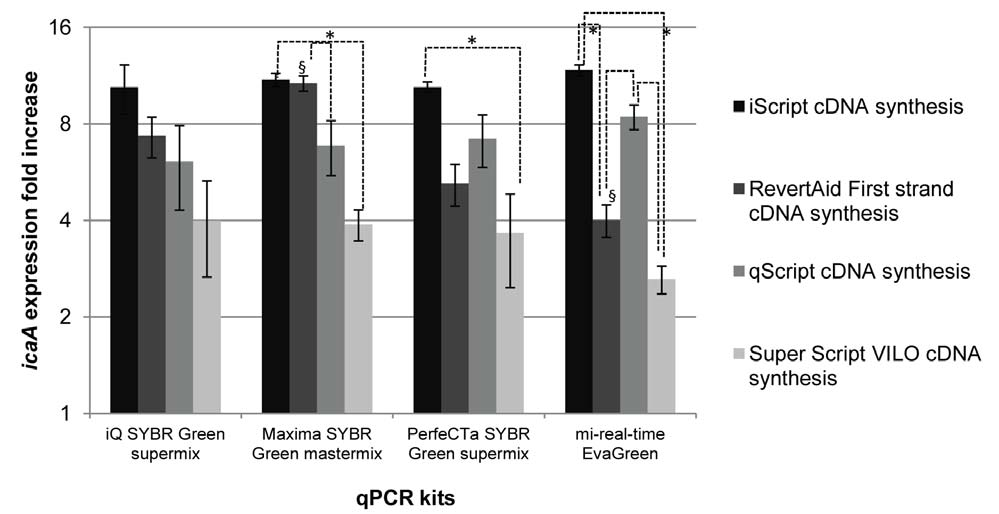
The impact of different cDNA and qPCR commercial kits in *icaA* gene expression quantification. cDNA from biofilms or planktonic cultures was synthesized using different kits. The impact of different qPCR master mixes in *icaA* quantification was also tested. The values represent the mean plus or minus standard deviation of 3 independent experiments. Statistical differences (p<0.05) between cDNA kits (*) or qPCR master mixes (§) were analyzed with ANOVA Tukey's test.

As different RNA extraction kits resulted in variable RNA quality, we also sought to determine whether the reverse transcriptase and qPCR kits would have similar variability. Therefore, the performance of different reverse transcriptase kits and qPCR master mixes commercially available were tested. Using the cDNA synthesized by different kits, *icaA* gene expression was quantified by qPCR, using distinct master mixes. Interestingly, as illustrated in Figure 2, significant differences were found in *icaA* mRNA levels when varying the reverse transcriptase kit (p<0.05, ANOVA), but not when varying the qPCR master mix (p>0.05, ANOVA). Despite the consistent *icaA* gene expression determination with different qPCR kits, the PCR efficiency varied somewhat: 84±4% for iQ™ SYBR® Green, 84±7% for Maxima ® SYBR Green 78±5% for PerfeCTa® SYBR® Green and 87±6% for mi-real time EvaGreen® master mixes. The efficiency of PerfeCTa® SYBR® Green was significantly different from the efficiency of mi-real-time EvaGreen® Master mix (p<0.05, ANOVA).

**Figure 3:**
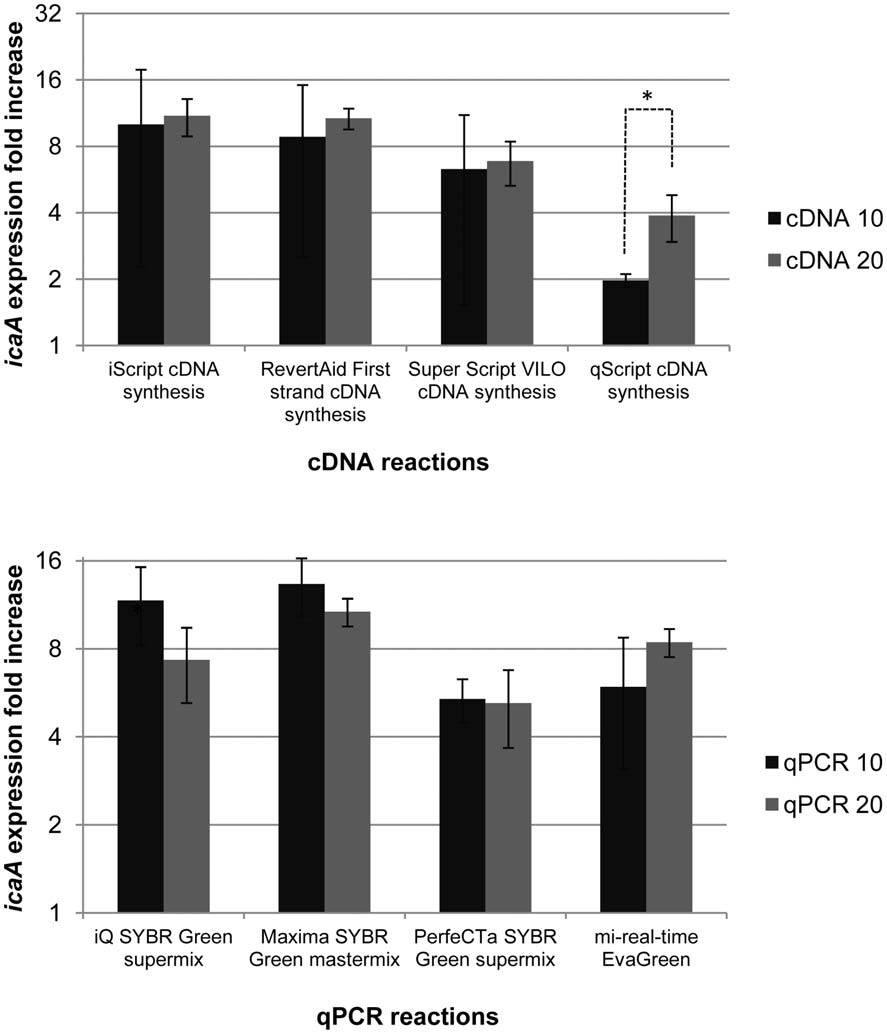
Variation in *icaA* gene expression quantification using different cDNA (top) or qPCR (botton) reaction volumes. TOP: cDNA, synthesized using 20 µL or 10 µL reaction volumes, was used for *icaA* transcripts quantification. The transcripts were detected using Maxima® SYBR Green Master Mix. BOTTOM: cDNA (1 100 dilution) synthesized using RevertAid™ First Strand cDNA synthesis kit (20 µL reaction) was used for *icaA* transcripts quantification by different qPCR master mixes and using different reaction volumes. The values represent the mean plus or minus standard deviation of 3 independent experiments. Statistical differences (*p<0.05) between 10 µL and 20 µL reactions were analyzed with paired t-test.

Reduction of the reverse transcriptase and qPCR volume reaction are among the possible ways to reduce costs associated with gene expression analysis. To determine if a lower volume of reaction can still provide consistent and reproducible results, reverse transcriptase reactions were performed using either 10 or 20 µL of volume and quantified with 20 µL volume reaction of Maxima® SYBR Green Master Mix. Simultaneously, cDNA obtained from a 20 µL reaction with RevertAid™ First strand cDNA synthesis kit was quantified using either 10 or 20 µL of qPCR reaction volume. As shown in Figure 3, the variation of qPCR volume did not affect the quantification of *icaA* gene expression (p>0.05, paired sample t-test). The same was not true for the reverse transcriptase reactions, since significant variation was found, particularly, in the cDNA obtained using SuperScript® VILO™ cDNA synthesis kit.

### Custom SYBR Green qPCR optimization

**Figure 4:**
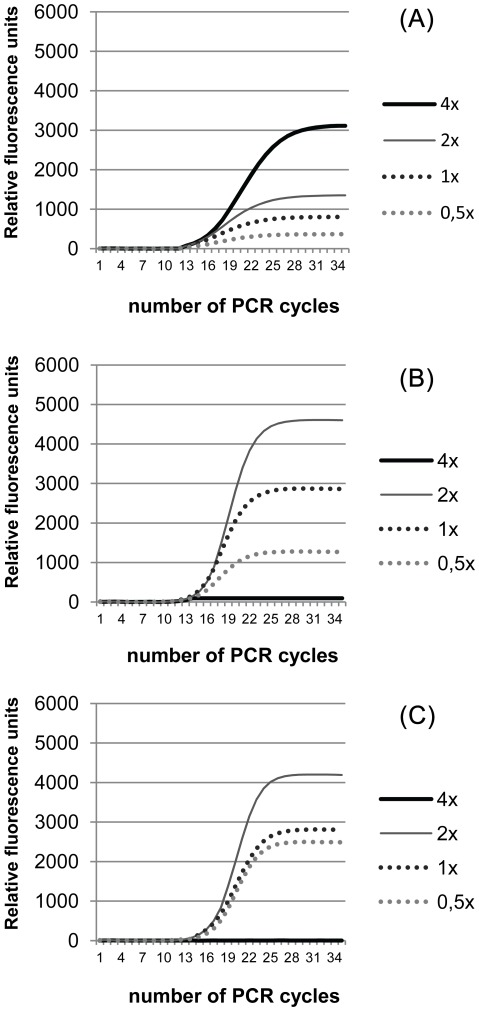
SYBR Green I dilution influence in qPCR assay using different Taq polymerase PCR kits. The tested SYBR Green I concentrations ranged from 0.5 to 4× using the following commercially available PCR kits: Ezyway Direct PCR Mix (top), MyTaq PCR mix (middle) and DyNAzyme™ II PCR Master Mix (bottom). Data shown is representative of two independent experiments.

Another option for reducing cost associated with gene expression analysis by qPCR is to prepare a custom SYBR Green qPCR mix. This can be achieved by using a common PCR mix (or the individual components of the mix, namely Taq polymerase + dNTPs + buffers) and adding the fluorescent dye. This approach requires several optimization steps, since SYBR Green I concentration can interfere with the PCR reaction [25], [26]. Using a 10,000× solution of SYBR Green I, different PCR mixes were titrated, ranging in final concentrations of SYBR Green I from 4× to 0.5×. As expected, SYBR Green I concentration strongly influenced the relative fluorescence units (RFU) detected in each reaction (Figure 4). Generally, the higher the concentration, the higher the RFU detected. However, in the custom mixes based on DyNAzyme™ II PCR Master Mix and MyTaq™ PCR, SYBR Green I concentrated 4× resulted in no RFU detection. To determine if this absence of RFU was result of any signal interference with the fluorescence detector or a PCR reaction inhibition, the products of the qPCR were run on a 1.5% agarose gel (Figure S2). It was observed that the absence of RFU in the qPCR mix with 4× SYBR Green I was the result of an effective inhibition of the PCR reaction.

### Validation of the custom qPCR gene expression assay

To validate the custom qPCR mix, we selected the DyNAzyme™ II PCR Master Mix supplemented with 1× SYBR Green I, and compared the outcome of gene expression to that obtained using Maxima ® SYBR Green Master Mix. We selected a set of genes that are known to be involved in biofilm formation and accumulation, namely *bhp, icaA*, and *aap* [27], [28], and also some genes involved in biofilm modulation, such as *agr* and *psmβ1* [11]. RNA was extracted from biofilm and planktonic cultures from four distinct *S. epidermidis* strains, that were previously characterized in terms of biofilm formation [21]. The cDNA used for the validation of the custom qPCR mix was synthesized with RevertAid™ First Strand cDNA synthesis kit in 20 µL reaction volume. No significant differences were found in PCR efficiency when using either custom or commercial mixes (88±7% for the custom assay). Additionally, the results obtained with the custom qPCR were consistent with the results obtained with the commercial Maxima® SYBR Green Master Mix, being either up- or down-regulated genes detected in similar quantities (Table 2). Nevertheless, statistically differences were found in 5 of the 20 comparisons (16, if excluded the genes that were not detected) (p<0.05, paired-samples t-test).

**Table 2:** Comparison of gene expression quantification using a custom qPCR mix and Maxima® SYBR Green Master Mix.

	*icaA*		*bhp*		*aap*		*psmβ1*		*agrB*	
Strain	Custom	Maxima®	Custom	Maxima®	Custom	Maxima®	Custom	Maxima®	Custom	Maxima®
**9142**	8,31±3,39*	12,87±2,51*	5,43±0,82*	7,02±1,11*	1,62±0,20*	2,14±0,32*	1,46±0,64	1,13±0,39	1,31±0,26	1,06±0,47
**IE186**	41,12±17,50	56,89±22,11	2,02±1,31	2,33±2,00	2,80±2,34	2,10±0,77	0,35±0,20	0,35±0,07	0,42±0,24	0,68±0,26
**M129**	45,63±21,14	71,53±59,28	N/D	N/D	5,81±1,04*	8,49±2,12*	0,19±0,14	0,21±0,11	0,99±0,94	1,19±0,21
**LE7**	6,40±2,42	5,50±3,33	N/D	N/D	3,47±0,74*	5,57±0,61*	0,48±0,34	0,31±0,11	0,60±0,36	0,77±0,09

cDNA (1 100 dilution**)** synthesized using a 20 µL reaction iScript™ cDNA synthesis and quantified in a 10 µL qPCR reaction using Maxima® SYBR Green Master Mix or custom made master mix. Values represent the fold difference between biofilm and planktonic population plus or minus standard deviation of 3 independent experiments. Values above 1 indicate up-regulation in biofilm, and below 1 indicates down-regulation.

* Statistical differences (p<0.05) between custom and Maxima® SYBR Green Master Mix reactions.

N/D – non detected.

## Discussion

The aim of this study was to optimize gene transcript quantification for *S. epidermidis* biofilm samples using qPCR. Optimization included minimization of time and cost, and maximization of reproducibility and sensitivity. Therefore, we addressed the three key steps of qPCR gene transcript analysis, namely RNA extraction, cDNA synthesis, and finally, the qPCR reaction.

RNA extraction, as a first step, is often considered the most important step, since poor quality RNA will unquestionably influence the reliability and reproducibility of the downstream applications [15]. Common indicators of RNA extraction success include the concentration , purity and integrity of RNA [29]. These indicators are influenced by both the sample's nature and the principle of the RNA extraction kit used [24], [30], [31]. Complex samples, such as *S. epidermidis* biofilms, are notoriously difficult to break up and the high content of proteins and polysaccharides in the biofilm matrix can interfere with downstream analysis, as we have shown recently [19], [20]. In that study, since FastRNA® Pro Blue was the only kit using mechanical lysis and had the highest performance, we tried to optimize the RNA extraction with the other kits tested (PureZOL™ from Bio-Rad and PureLink™ from Invitrogen) by performing the mechanical lysis step of the FastRNA® Pro Blue kit and using the lysis buffers included with the respective kits. However, this modification did not significantly increase the RNA yield [19], suggesting that the high efficiency of FastRNA® Pro Blue was not only due to the mechanical lysis, but also due to the chemical composition of the buffer. We have reported similar results for other bacterial species that form biofilms, such as *Listeria monocytogenes* [20].

By analyzing FastRNA® Pro Blue buffer composition, we devised the custom procedure described here, wherein 90% phenol solution was added to the buffer of each silica-based membrane commercial kits in a proportion of 1 1. This approach significantly increased the RNA yield, with no detectable reduction of RNA purity and integrity. To see if other commercial silica-based column kits could also be used with this approach, three other kits were successfully tested. Of note, the FavorPrep™ kit was originally optimized to RNA extraction from human cells, but was easily adapted for bacterial cultures.

The custom protocols did not surpass the FastRNA® Pro Blue kit in terms of RNA quality or yield; however, we also evaluated time necessary to perform the protocol and the cost associated (Table 3 and Table S2). In comparison with FastRNA® Pro Blue kit, we could achieve a 68% cost reduction, per reaction, when using our custom RNA protocol. Furthermore, the overall experiment took us only 40 minutes to process 6 samples, versus nearly 4 hours with the FastRNA® Pro Blue. Furthermore, the custom RNA extraction also reduced the operator exposure to hazardous substances such as chloroform, used in RNA organic extractions, and β-mercaptoethanol, which is regularly used as adjuvant for bacterial cell lysis in column-based extractions.

**Table 3:** Analysis of the percentage of cost reduction when using the custom RNA extraction and qPCR instead of the commercially available kits.

		Kit	Price/reaction (€)	% of cost reduction
RNA extraction	Commercial	FastRNA® Problue	7.15	N/A
		Based on PureLink™	5	30%
	Custom	Based on ISOLATE™	4.2	41%
		Based on Direct-Zol™	4	44%
		Based on FavorPrep™	2.3	68%
qPCR assay	Commercial	Maxima ® SYBR Green Master Mix	0.48	N/A
	Custom	DyNAzyme™ II PCR Master Mix+SYBR GreenI	0.13	73%

The price per reaction already includes all the extra-reagents needed. Additionally, the values presented here and the comparison performed are relative to the price of largest kit available on the market.

N/A - not applicable.

Without questioning the importance of RNA extraction step, a previous study regarding the optimization of cDNA synthesis using commercially available kits, revealed a high variability in the results obtained by some of the kits tested, indicating that the reverse transcriptase reaction is also crucial in order to obtain reliable measurement of mRNA transcripts [32]. Our results have confirmed the observations of Sieber *et al.* [32], in that a high variability was found in the quantification of cDNA obtained with different synthesis kits. Curiously, no significant variation was found in the reverse transcriptase kits when quantified by the iQ™ SYBR® Green Supermix (p>0.05, ANOVA).

The presence of PCR inhibitors in the cDNA was tested by serial dilution of the samples. Using the 2^ΔCt^ method, a variation of the Livak method [33], if no PCR inhibitors are present in the cDNA, the relative fold increase of a specific gene should remain constant as cDNA is diluted (assuming a reasonable dilution range) [29]. While this was true for some cDNA synthesis kits, there were others that clearly yielded product containing PCR inhibitors. By using 100 fold cDNA dilution, we found that regardless the qPCR master mix used, no significant variation in gene expression quantification was detected, even when using different cDNA sources. In the Sieber *et al.* study, besides the reverse transcriptase variability, they also reported, although lower, some variability associated with the qPCR kit used [32]. Actually, the majority of the qPCR master mixes tested here, including the custom qPCR mix, presented similar efficiencies (85±6% average) with the exception of the PerfeCTa® SYBR Green SuperMix (78±5%) (p<0.05, ANOVA).

While many qPCR kits recommend the use of 50 µL reactions, we previously reduced the volume to 25 µL and were able to properly detect gene expression both in *E. coli* [34] and *S. aureus* [35]. The reduction of reaction volume is appealing, as it reduces the costs associated with an experiment. However, smaller volumes can introduce more pipetting errors and may reduce the limit of detection. To address this issue, reverse transcriptase and qPCR reactions were performed in either 10 or 20 µL volumes (Figure 3). Interestingly, no significant differences were found between 10 or 20 µL qPCR reactions in any of the tested kits. On the other hand, in the cDNA synthesis kits tested, the variation was higher in 10 µL reverse transcriptase reactions, as noted by the higher standard deviation presented. (Figure 3).

The reduction of either cDNA or qPCR volume reaction from 20 to 10 µL, will unquestionably allow the reduction of some of the costs associated with gene expression analysis. Nevertheless, regarding the cDNA synthesis, we observed, in some particular cases, significant variability associated with reduced volume reactions. This higher variability would require an increase in the number of technical replicates in order to obtain reliable and meaningful results. Therefore, in our judgment, the reduction of the reverse transcriptase volume reactions might not be advantageous and ultimately, might not reduce overall costs (Table S3).

Contrary to the reverse transcriptase reaction, a reduction in the qPCR volume reaction was not associated with changes in the outcome of the experimental assay. Therefore, the use of 10 µL volume reaction instead of the 25 or 50 µL recommended by the manufacturer's will allow to perform between 2.5 to 5 more reactions with the same cost (Table S4).

A further approach to reduce experimental costs is to add SYBR Green I to a PCR mix, as such mixes are often considerably cheaper than qPCR mixes (Table S4). A fundamental step to be taken in consideration is an initial titration of the SYBR Green I, as a concentration can diminish the sensitivity and limit of detection and a higher concentration can inhibit the PCR reaction, as shown in our results. According to Figure 4, a titration of 0.5× SYBR Green I in DyNAzyme™ II PCR Master Mix, would be sufficient to detect the PCR products. However, as qPCR's done using Maxima ® SYBR Green Master Mix would yield RFU levels of around 4000–5000, we decided to use the 1× SYBR Green I concentration (since no PCR inhibition was detected) in order to obtain similar RFU levels.

To validate our protocol, RNA from planktonic cultures and biofilms from 4 different *S. epidermidis* strains was extracted and analyzed as described. The criteria considered upon evaluation of the kits included not only reproducibility and accuracy of the experiments but also the time and costs associated (Figure S3). Several known genes involved in *S. epidermidis* biofilm formation, accumulation and modulation were selected as a control since their function and expression levels have been widely studied [6], [36]–[41]. Both commercial and custom master mix detected the expected gene transcript levels in *S. epidermidis* biofilms when compared with planktonic cultures, validating our custom qPCR master mix. Despite the statistically significant differences (p<0.05, paired-samples t-test) found between the commercial and the custom qPCR master mixes in the expression of *S. epidermidis* 9142 *icaA*, *bhp* and *aap*, in *S. epidermidis* M129 *aap* and *S. epidermidis* LE7 *aap*, these differences had no biological significance. All the differences were below 1.8 fold (average difference between custom and commercial kit was 1.48±0.18 fold), meaning an average difference in cycle threshold variation of 0.5 PCR cycles. Furthermore, both increases and decreases in transcript levels were detected in both experimental setups. Since the overall priming efficiency of the custom qPCR was similar to the commercial kits, we hypothesize that those small differences could be the result of variations in each SYBR Green I titration of the standard PCR mix, as we have detected some batch to batch variations in RFU's and PCR efficiencies. As the initial cost of SYBR Green I is high, it can be used in other applications, such as agarose gel DNA/RNA staining. Once we thaw the aliquots, we kept them at 4°C, protected from light. We did not address the effect of storing SYBR Green I at 4°C, although the manufacturer suggests that short-term storage is possible. For future reference, smaller aliquots should be prepared, so that freshly thawed SYBR Green I could be used.

The qPCR custom master mix developed in this study not only produced comparable results to those obtained using commercially available master mixes, it also, allowed considerable reduction in the cost associated with gene expression quantification, around 70% (Table 3 and Table S4).

Currently, qPCR is considered the gold standard technique to study transcript levels of a specific set of genes and to validate the results obtained in genome-wide analysis such as DNA microarrays and RNA sequencing. Therefore, qPCR is a technique in high demand that has to assure high reliability, sensitivity and reproducibility. Herein, we describe a custom procedure for RNA extraction and qPCR analysis that present the same high standards as the commercially available and reduces the high costs normally associated with gene expression analysis.

## Supporting Information

Figure S1 **RNA integrity determined by visualization in ethidium bromide stained agarose gel.** DL - DNA ladder (23 Kbp), C1- Custom w/PureLink™ Mini Kit; C2- Custom w/FavorPrep™ Blood/Cultured cell total RNA; C3- Custom w/Direct-zol™ RNA MiniPrep; C4- Custom w/ISOLATE RNA Mini kit; PL- PureLink™; PB – FastRNA® Pro Blue.
(TIF)

Figure S2 **Effect of SYBR Green I concentration in the inhibition of the qPCR.** The qPCr was performed using the DyNAzyme™ II PCR Master Mix.
(TIF)

Figure S3 **Workflow chart used to compare the performance of the RNA extraction procedures, cDNA synthesis kits and qPCR master mixes tested.**
(TIF)

Table S1 Oligonucleotide primer sequences.
(DOC)

Table S2 Kits and reagents used for the RNA extraction. All the prices listed were obtained by quote during January 2012.
(DOC)

Table S3 cDNA synthesis kits used and price per reaction. All the prices listed were obtained by quote during January 2012.
(DOC)

Table S4 qPCR kits and reagents used and prices per reaction. All the prices listed were obtained by quote during January 2012 * kit to which SYBR Green I was added.
(DOC)
