# Supplementary figures and images for: Optimizing a qPCR Gene Expression Quantification Assay for S. epidermidis Biofilms: A Comparison between Commercial Kits and a Customized Protocol
Source: PLoS One. 2012 May 21;7(5):e37480. doi: 10.1371/journal.pone.0037480 (PMC3357405; doi:10.1371/journal.pone.0037480)

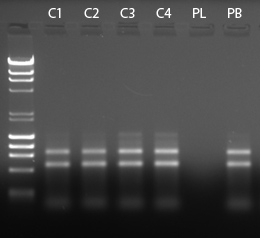

Supplement: Figure S1 — RNA integrity determined by visualization in ethidium bromide stained agarose gel. DL - DNA ladder (23 Kbp), C1- Custom w/PureLink™ Mini Kit; C2- Custom w/FavorPrep™ Blood/Cultured cell total RNA; C3- Custom w/Direct-zol™ RNA MiniPrep; C4- Custom w/ISOLATE RNA Mini kit; PL- PureLink™; PB – FastRNA® Pro Blue. (TIF) [file pone.0037480.s001.tif]

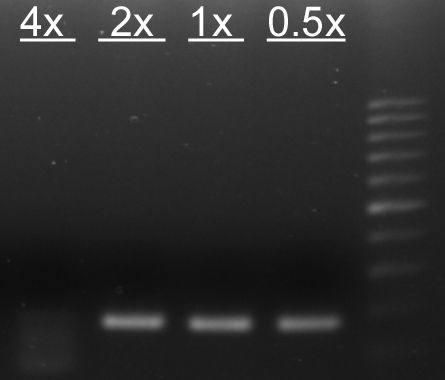

Supplement: Figure S2 — Effect of SYBR Green I concentration in the inhibition of the qPCR. The qPCr was performed using the DyNAzyme™ II PCR Master Mix. (TIF) [file pone.0037480.s002.tif]

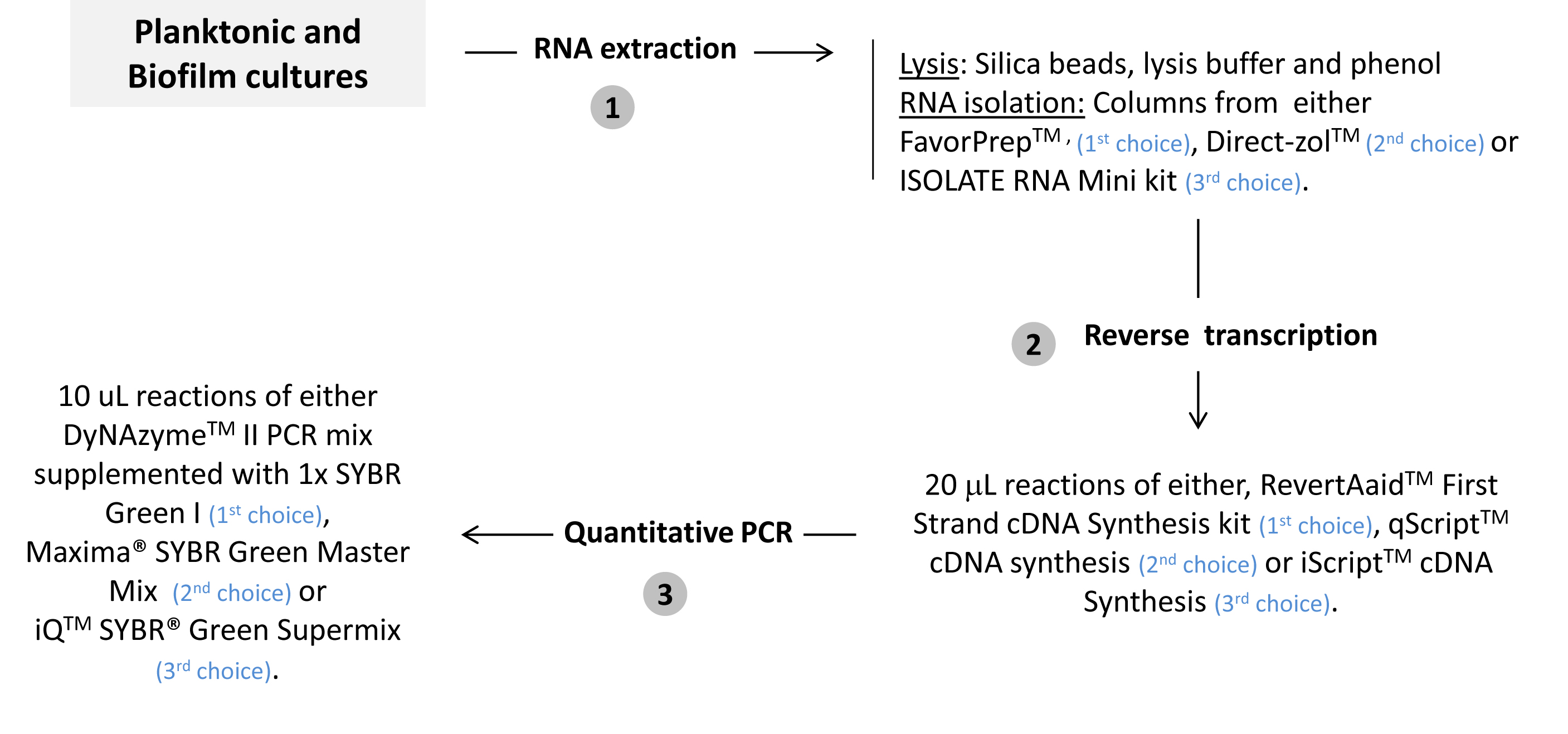

Supplement: Figure S3 — Workflow chart used to compare the performance of the RNA extraction procedures, cDNA synthesis kits and qPCR master mixes tested. (TIF) [file pone.0037480.s003.tif]
