# Supplementary material for: Optimizing a qPCR Gene Expression Quantification Assay for S. epidermidis Biofilms: A Comparison between Commercial Kits and a Customized Protocol
Source: PLoS One. 2012 May 21;7(5):e37480. doi: 10.1371/journal.pone.0037480 (PMC3357405; doi:10.1371/journal.pone.0037480)
Supplement: Table S1 — Oligonucleotide primer sequences. (DOC) [file pone.0037480.s004.doc]

**Supplementary Table S 1. Oligonucleotide primer sequences.**

| **Target gene** |  | **Oligonucleotide primers sequence (5’ to 3’)** | **TM**  **(^o^C)** | **Amplicon size (bp)** |
| --- | --- | --- | --- | --- |
| ***16S*** | FW | gggctacacacgtgctacaa | 59.79 | 176 |
|  | RV | gtacaagacccgggaacgta | 59.85 |  |
| ***icaA*** | FW | tgcactcaatgagggaatca | 60.20 | 134 |
|  | RV | taactgcgcctaattttggatt | 59.99 |  |
| ***aap*** | FW | gcaccagctgttgttgtacc | 59.22 | 190 |
|  | RV | gcatgcctgctgatagttca | 59.98 |  |
| ***bhp*** | FW | tggactcgtagcttcgtcct | 60.01 | 213 |
|  | RV | tctgcagatacccagacaacc | 60.13 |  |
| ***agrB*** | FW | AATTCGTTTAGGGATGCAGGT | 59.85 | 142 |
|  | RV | ACCGTGTGCATGTCTCCTAAT | 59.49 |  |
| ***psmβ1*** | FW | AGCAGAAGCTATTGCAAATACAG | 57.96 | 105 |
|  | RV | CCTAATACGCTAACGCCACTTT | 59.72 |  |

**Supplementary Table S 2. Kits and reagents used for the RNA extraction.** All the prices listed were obtained by quote during January 2012.

| **Kit (Manufacturer)** | **Number of extractions per kit** | **prices (€) per reaction** |
| --- | --- | --- |
| FastRNA® Pro Blue (MPBiomedicals) | 50 | 7,14 |
| PureLink^TM^ RNA Mini Kit (Invitrogen) | 10-50 | 9,70-4,46 |
| ISOLATE RNA Mini Kit (Bioline) | 10-250 | 5,30-3,68 |
| Direct-zol^TM^ RNA MiniPrep (Zymo Research) | 50-200 | 4,19-3,41 |
| FavorPrep Blood/Cultured Cell Total RNA (Favorgen) | 50-300 | 2,50-1,80 |
| Ethanol 100% (Fisher) | 2500* | 0,006 |
| Chloroform (Fisher) | 3333-8333 | 0,002-0,001 |
| Phenol (AppliChem) | 277-1387 | 0,08-0,06 |
| Glass beads, acid-washed, 150-212 μm (Sigma) | 25-1250 | 1,53 – 0,33 |
| RNAse & DNAse free tubes with screwcap (BioPlastics) | 500 | 0,12 |

* ethanol is used on variable volume. An overestimated 1 mL volume was used for the purpose of price calculations.

**Supplementary Table S 3. cDNA synthesis kits used and price per reaction.** All the prices listed were obtained by quote during January 2012.

| **Kit (manufacturer)** | **Number of 20 μL reactions per kit** | **Prices (€) per reaction** |
| --- | --- | --- |
| SuperScript® VILO^TM^ synthesis (Invitrogen) | 50-250 | 10,08-8,88 |
| RevertAid^TM^ First Strand cDNA synthesis (Fermentas) | 20-100 | 4,25-2,80 |
| iScript^TM^ cDNA synthesis (Bio-Rad) | 25-100 | 6,48-4,97 |
| qScript^TM^ cDNA synthesis (Quanta BioSciences) | 25-500 | 4,52-3,04 |

**Supplementary Table S 4. qPCR kits and reagents used and prices per reaction.** All the prices listed were obtained by quote during January 2012 * kit to which SYBR Green I was added.

| **Kit (Manufacturer)** | **Number of 20 μL reactions per kit** | **Prices (€) per reaction** |
| --- | --- | --- |
| mi-real-time EvaGreen® Master (Metabion) | 250-1250 | 0,44-0,35 |
| Maxima ® SYBR Green Master Mix (Fermentas) | 250-5000 | 0,81-0,48 |
| iQ^TM^ SYBR® Green Supermix (Bio-Rad) | 250-5000 | 0,77-0,59 |
| PerfeCTa® SYBR® Green SuperMix (Quanta BioSciences) | 250-5000 | 0,70-0,58 |
| DyNAzyme^TM^ II PCR Master Mix (Finnzymes)* | 500-2500 | 0,12-0,11 |
| MyTaq^TM^ Mix (Bioline)* | 500-2500 | 0,21-0,18 |
| EzWay Direct PCR Master Mix (Koma Biotech)* | 100 | 1,40 |
| SYBR green I nucleic acid gel stain (Invitrogen) | 12500-25000 | 0,03-0,02 |
