# Supplementary material for: Optimizing a qPCR Gene Expression Quantification Assay for S. epidermidis Biofilms: A Comparison between Commercial Kits and a Customized Protocol
Source: PLoS One. 2012 May 21;7(5):e37480. doi: 10.1371/journal.pone.0037480 (PMC3357405; doi:10.1371/journal.pone.0037480)
Supplement: Table S2 — Kits and reagents used for the RNA extraction. All the prices listed were obtained by quote during January 2012. (DOC) [file pone.0037480.s005.doc]

**Supplementary Table S 2. Kits and reagents used for the RNA extraction.** All the prices listed were obtained by quote during January 2012.

| **Kit (Manufacturer)** | **Number of extractions per kit** | **prices (€) per reaction** |
| --- | --- | --- |
| FastRNA® Pro Blue (MPBiomedicals) | 50 | 7,14 |
| PureLink^TM^ RNA Mini Kit (Invitrogen) | 10-50 | 9,70-4,46 |
| ISOLATE RNA Mini Kit (Bioline) | 10-250 | 5,30-3,68 |
| Direct-zol^TM^ RNA MiniPrep (Zymo Research) | 50-200 | 4,19-3,41 |
| FavorPrep Blood/Cultured Cell Total RNA (Favorgen) | 50-300 | 2,50-1,80 |
| Ethanol 100% (Fisher) | 2500* | 0,006 |
| Chloroform (Fisher) | 3333-8333 | 0,002-0,001 |
| Phenol (AppliChem) | 277-1387 | 0,08-0,06 |
| Glass beads, acid-washed, 150-212 μm (Sigma) | 25-1250 | 1,53 – 0,33 |
| RNAse & DNAse free tubes with screwcap (BioPlastics) | 500 | 0,12 |

* ethanol is used on variable volume. An overestimated 1 mL volume was used for the purpose of price calculations.
