# Supplementary material for: Optimizing a qPCR Gene Expression Quantification Assay for S. epidermidis Biofilms: A Comparison between Commercial Kits and a Customized Protocol
Source: PLoS One. 2012 May 21;7(5):e37480. doi: 10.1371/journal.pone.0037480 (PMC3357405; doi:10.1371/journal.pone.0037480)
Supplement: Table S3 — cDNA synthesis kits used and price per reaction. All the prices listed were obtained by quote during January 2012. (DOC) [file pone.0037480.s006.doc]

**Supplementary Table S 3. cDNA synthesis kits used and price per reaction.** All the prices listed were obtained by quote during January 2012.

| **Kit (manufacturer)** | **Number of 20 μL reactions per kit** | **Prices (€) per reaction** |
| --- | --- | --- |
| SuperScript® VILO^TM^ synthesis (Invitrogen) | 50-250 | 10,08-8,88 |
| RevertAid^TM^ First Strand cDNA synthesis (Fermentas) | 20-100 | 4,25-2,80 |
| iScript^TM^ cDNA synthesis (Bio-Rad) | 25-100 | 6,48-4,97 |
| qScript^TM^ cDNA synthesis (Quanta BioSciences) | 25-500 | 4,52-3,04 |
