# Supplementary material for: Optimizing a qPCR Gene Expression Quantification Assay for S. epidermidis Biofilms: A Comparison between Commercial Kits and a Customized Protocol
Source: PLoS One. 2012 May 21;7(5):e37480. doi: 10.1371/journal.pone.0037480 (PMC3357405; doi:10.1371/journal.pone.0037480)
Supplement: Table S4 — qPCR kits and reagents used and prices per reaction. All the prices listed were obtained by quote during January 2012 * kit to which SYBR Green I was added. (DOC) [file pone.0037480.s007.doc]

**Supplementary Table S 3. qPCR kits and reagents used and prices per reaction.** All the prices listed were obtained by quote during January 2012 * kit to which SYBR Green I was added.

| **Kit (Manufacturer)** | **Number of 20 μL reactions per kit** | **Prices (€) per reaction** |
| --- | --- | --- |
| mi-real-time EvaGreen® Master (Metabion) | 250-1250 | 0,44-0,35 |
| Maxima ® SYBR Green Master Mix (Fermentas) | 250-5000 | 0,81-0,48 |
| iQ^TM^ SYBR® Green Supermix (Bio-Rad) | 250-5000 | 0,77-0,59 |
| PerfeCTa® SYBR® Green SuperMix (Quanta BioSciences) | 250-5000 | 0,70-0,58 |
| DyNAzyme^TM^ II PCR Master Mix (Finnzymes)* | 500-2500 | 0,12-0,11 |
| MyTaq^TM^ Mix (Bioline)* | 500-2500 | 0,21-0,18 |
| EzWay Direct PCR Master Mix (Koma Biotech)* | 100 | 1,40 |
| SYBR green I nucleic acid gel stain (Invitrogen) | 12500-25000 | 0,03-0,02 |
